# Lower grip strength and insufficient physical activity can increase depressive symptoms among middle-aged and older European adults: a longitudinal study

**DOI:** 10.1186/s12877-022-03392-x

**Published:** 2022-08-22

**Authors:** Han Zheng, Qingwen He, Hongyan Xu, Xiaowei Zheng, Yanfang Gu

**Affiliations:** 1 Department of Public Health, the Affiliated Wuxi Maternity and Child Health Care Hospital of Nanjing Medical University, 48 Huaishu Road, Liangxi District, Wuxi, Jiangsu 214002 P.R. China; 2 Department of Public Health and Preventive Medicine, Wuxi School of Medicine Jiangnan University, 1800 Lihu Road, Binhu District, Wuxi, Jiangsu 214122 P.R. China

**Keywords:** Grip strength, Physical inactivity, Depression, Older people

## Abstract

**Objectives:**

The present study aimed to identify the gender-specific trajectories of grip strength using group-based trajectories, explore the interaction between grip strength and physical activity on depression, and investigate the association of physical activity with the change in depression by different grip strength groups among middle-aged and older European adults.

**Methods:**

A total of 14,098 participants aged 50 years or older from the Survey of Health, Ageing and Retirement in Europe 2007–2019 were included in this study. Group-based trajectory modeling was used to identify the low, middle and high group of grip strength by gender. Generalized estimated equations were fitted to analyze the interaction effect. The data of wave 2-wave 5 and wave 2-wave 7 were chosen to conduct sensitivity analyses.

**Results:**

Significant interactions between grip strength group and physical inactivity were found (*x*^2^_interaction_ = 11.16, *P* = 0.004). Significant interactions between physical inactivity and time on depression were identified in low (*x*^2^_interaction_ = 27.83, *P* < 0.001) and moderate (*x*^2^_interaction_ = 23.67, *P* < 0.001) grip strength, but a similar result was not found in high grip strength (*x*^2^_interaction_ = 4.39, *P* = 0.495). Participants in the physical inactivity group had higher depression scores in the low and moderate grip strength groups. Sensitivity analyses yield almost similar results.

**Conclusions:**

Grip strength and physical inactivity interact with depression. Lower grip strength and insufficient physical activity can increase depressive symptoms. People with lower grip strength and physical inactivity should pay special attention to the prevention of depression.

**Supplementary Information:**

The online version contains supplementary material available at 10.1186/s12877-022-03392-x.

## Background

Depression is the leading cause of mental health disorders worldwide, affecting an estimated 300 million people [1]. Meanwhile, the increasing aging population and elongated life expectancy make it a great challenge for humans [2]. Late-life depression is highly prevalent in aging people, and is a difficult challenge. Moreover, late-life depression dramatically reduces the quality of life [3]. Long-term depression increases morbidity risk [4], mortality [5] and the frequency of healthcare service use [6]. Multiple factors, including dysfunctional cognitions, stressful life events, health status and interpersonal dysfunction, are significantly associated with depression [7, 8]. In addition, it has been reported that higher handgrip strength and sufficient physical activity are negatively related to depression, they could improve mental health [9, 10].

Handgrip strength is a well-established measure of physical performance/muscle strength and has been widely used in an observational cohort studies and clinical settings [11, 12]. Numerous studies have shown that handgrip strength is associated with depressive symptoms, and individuals with higher handgrip strength tend to have a lower risk of depression [9, 13]. In addition, evidence of sex-related differences in handgrip strength that decrease with age has been reported [14]. However, the definition of good grip strength remians controversial [15]. Most studies group handgrip strength use the percentage method based on data distribution [16]. The time effect on grip strength is ignored, although the above studies have considered the influence of sex and age on grip strength.

The relationship between physical activity and handgrip strength has been reported in previous studies. Both studies recommend physical activity during middle age to protect against sarcopenia and depression when the handgrip strength starts to decline [17, 18]. Furthermore, engagement in physical activity has a psychological benefit in later life [19]. However, to our knowledge, little information is currently available on the combined effects of grip strength and physical activity on depression in older people and whether the effect of physical and long-term changes on depression is differentiated by grip strength groups.

Hence, we conducted a prospective study with a large, multinational cohort derived from the Survey of Health, Ageing and Retirement in Europe (SHARE) 2005–2019 to (1) identify the gender-specific trajectories of grip strength using group-based trajectories (GBTM) [20]; (2) explore the joint effect of grip strength trajectory groups and physical inactivity, and (3) investigate the association of insufficient physical activity with a long-term change in depression according to grip strength.

## Methods

### Study population

Data were obtained from SHARE, which is biennial longitudinal study aimed at assessing the population aged ≥50 years across European countries using probability-based sampling. Details of the sampling methodology can be found in an official published article [21]. The information in this survey concerned health, socioeconomic status, and social and family networks. Seven waves (1, 2, 4, 5, 6, 7, and 8) and one retrospective life history wave (3) have been conducted since 2004. Based on the first wave, SHARE has successively incorporated more European countries since the second wave, including the Czech Republic, Poland, and Ireland. Thus, we analyzed data from six-panel waves of SHARE but excluded the first and third waves in the present study. SHARE was reviewed and approved by the ethics committee of the University of Mannheim and the Ethics Council of the Max Planck Society.

**Fig. 1:**
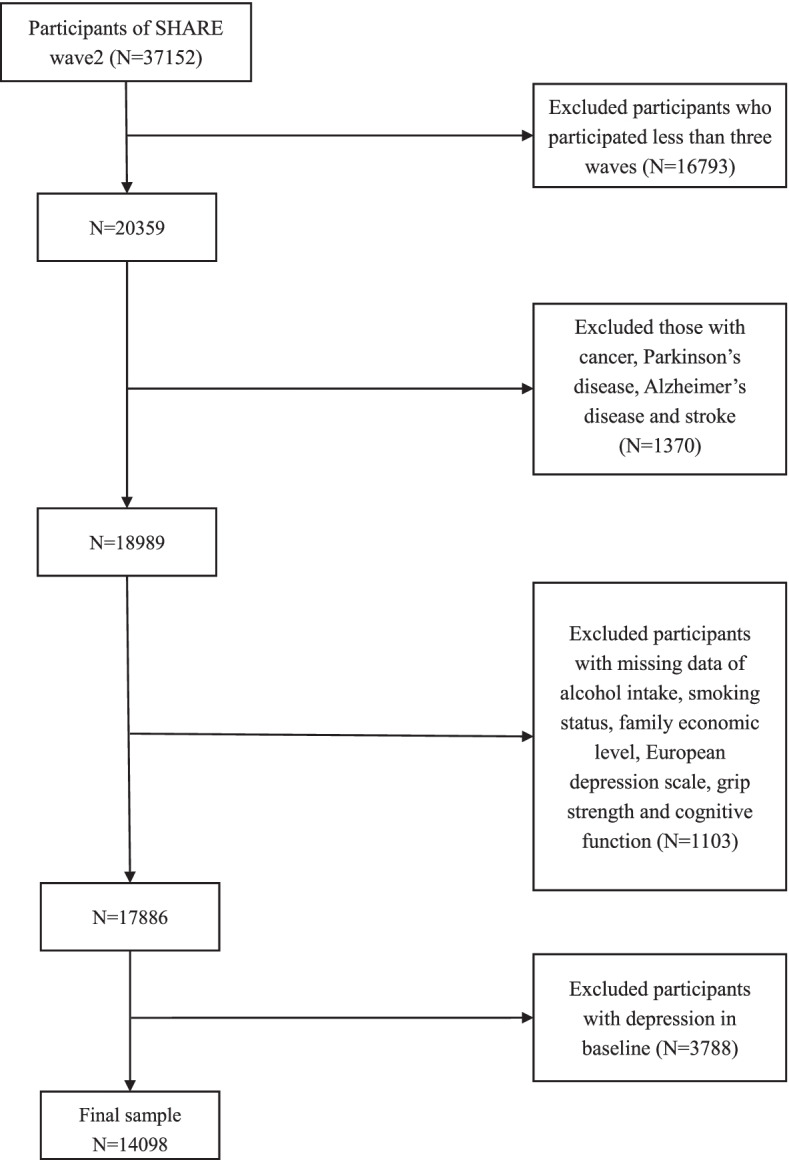
Flow chart of the analytic sample

A total of 37,152 people participated in the second wave, which was conducted in 2006. The exclusion criteria were as follows: (1) participation in less than three waves during the following years (*n* = 16,793); (2) those with cancer, Parkinson’s disease, Alzheimer’s disease, and stroke in any survey that may affect handgrip strength measurement (*n* = 1370); (3) those with missing data on alcohol intake, smoking status, family economic level, European depression scale, grip strength and cognitive function (*n* = 1103) and (4) individuals with depression at baseline (wave 2) to avoid causing reverse causality (*n* = 3788). Ultimately, 14,098 individuals were included in this study. The selection process for the study population is shown in Fig. 1. Most baseline characteristics were well balanced between the original wave 2 (excluding subjects without studied variables and with baseline depression) and the final analyzed wave 2 participants, indicating that those enrolled participants represented the total participants (S**-**Table 1).

### Depression

Depression was evaluated using the European Depression Scale (EURO-D). The scale is a 12-item binary scale that includes the following symptoms: depression, pessimism, suicidality, guilt, sleep, irritability, fatigue, appetite, interests, enjoyment, concentration and tearfulness, and has been validated by the European Depression Concerted Action Project [22]. The total EURO-D scale ranged from 0 to 12, with a score above three representing depression [23].

### Physical inactivity

Physical activity was assessed by asking two questions about how often they engage in moderate- and vigorous-intensity physical activities daily. The following response options were provided: more than once a week, once a week, one to three times a month, and hardly ever or never. Participants who reported ‘hardly ever or never’ for both moderate- and vigorous-intensity physical activity were defined as physical inactivity [24].

### Grip strength

Grip strength was measured using a Smedley handheld dynamometer (100 kg) [25]. Participants were asked to sit or stand while keeping their upper arms tight against the trunk with their elbows at a 90° angle and then squeeze the handles as hard as possible for 5 s. Two alternate measures from their right and left hands were performed, the highest value of four measures in each survey wave was used in the present study. Due to the lack of a standard grouping method for grip strength, we used GBTM to explore a suitable group according to sex based on the panel data.

### Covariates

Covariates in this study were acquired from the questionnaire, including age, gender (male/female), European region (central, northern, southern, and western Europe), marital status (married, living with a spouse, or other married status such as divorced, widowed), education (primary/secondary/tertiary education). Furthermore, employment status (employed/retired/unemployment), family economic level, smoking status (never, ever, and current smoker), and alcohol intake (drinking exceeds two glasses) were also acquired. In addition, heart attack (yes/no), hypertension (yes/no), hyperlipidemia (yes/no), diabetes (yes/no), mobility limitation (yes/no), body mass index (BMI), and cognitive function were acquired. Education was categorized according to the International Standard Classification of Education [26]. The family economic level was determined by one question: “Is your household able to make ends meet?” The answers included easy, fairly easy, with some difficulty, and with great difficulty. BMI was calculated as weight in kilograms divided by height in meters squared. Cognitive function was assessed in four domains: time orientation, memory, verbal fluency, and numeracy [27, 28]. The scores for each domain ranged from 0 to 5, 0–20, 0–100, and 0–5, respectively. To avoid the proportion of memory and fluency being too high, we standardized the four scores and the sum of the these scores were used to assess cognitive function.

### Statistical analysis

All statistical analyses were conducted using STATA version 16.0 (Stata Corp, College Station, Texas, USA). A two-stage approach is used to select the optimal number of groups and trajectory shapes. First, we fitted all trajectory models in cubic form, initiated a model with one trajectory, and then fitted the models up to the optimal number of trajectories based on the Bayesian information criterion value (BIC). The minimum sample size for each trajectory was > 5%. The average posterior probability of assignments (APPA) values should be> 70%, and the odds of correct classification (OCC) should be> 5.0. We examined models based on all grip strength data and found three trajectory groups based on BIC values that appeared to provide the best balance between data fit and complexity. GBTM identified low-, moderate-, and high-grip strength groups of individuals following similar patterns of grip strength according to gender. Data are presented as mean and standard deviation (SDs) for continuous variables or as percentages for categorical variables. One-way analysis of variance (ANOVA) was used to examine the difference in means for continuous variables with normal distribution; otherwise, the Kruskal-Wallis test was used. Pearson’s χ2 test was performed to compare the distribution of categorical variables among the three grip strength groups. Multiplicative interaction was assessed using the grip strength-physical inactivity interaction term in the generalized estimated equation (GEE) model. After determining the joint effect of grip strength and exercise on depression, the time variable interacted with physical inactivity according to the grip strength group to identify the independent effect of exposure on the change in depression over time. Depression was treated as a continuous variable in all the GEE models. The independent working correlation structure was chosen for the GEE analysis.

Considering that the majority of participants were lost to follow-up due to the relatively long survey time of SHARE, we further performed sensitivity analyses with.

data from waves 2 to 5 and from waves 2 to 7 (all excluding wave 3) of SHARE to ensure the robustness of the results.

All GEE models were adjusted for potential confounders including age, gender, European region, marital status, education, employment status, family economic level, smoking status, alcohol intake, heart attack, hypertension, hyperlipidemia, diabetes, mobility limitation, BMI and cognitive function. A two-tailed *P* value≤0.05 was recognized as statistically significant.

## Results

### Baseline characteristics of participants

This study included 3123 participants in the low grip strength group, 7454 in the moderate grip strength group, and 3521 in the high grip strength group. The gender-specific grip strength groups explored using GBTM are presented in S**-**Fig. 1. Grip strength trajectories met the model evaluation criteria (S**-**Table 6). The mean follow-up time was 9.25 years. The baseline characteristics of the participants across the three grip strength groups are shown in Table 1. Individuals with high grip strength tended to be younger (mean age, 58.12 years old), male (48.08%), married (80.26%), had secondary education (56.43%), had a higher family economic level, had higher alcohol intake, have a higher BMI (average BMI, 26.91 kg/m^2^) and had cognitive function (average standardized score, 2.14). Participants with low grip strength were more likely to live in Southern Europe (35.34%), retired (67.92%), never smoked (41.63%), had a heart attack (12.74%), had hypertension (39.39%), had hyperlipidemia (24.78%), had diabetes (14.22%), had mobility limitation (58.70%), and were physically inactive (9.54%). The average depression scores increased over time, but this trend was more obvious in the low and moderate grip strength groups than in the high grip strength group.

**Table 1:** Baseline characteristics of participants according to grip strength groups

	Low grip strength (*n* = 3123)	Middle grip strength (*n* = 7454)	High grip strength (*n* = 3521)	*P*
Age^a^	70.69 ± 8.39	63.26 ± 7.40	58.12 ± 5.44	< 0.001^*^
Gender (%)^b^				
Female	53.38	50.99	48.08	< 0.001^*^
Region (%)^b^				
Central Europe	29.23	33.16	31.41	< 0.001^*^
Northern Europe	12.97	17.05	24.34	
Southern Europe	35.34	25.17	17.27	
Western Europe	21.45	24.62	26.98	
Married Status (%)^b^				
Married, living with a spouse	68.97	76.43	80.26	< 0.001^*^
Education (%)^b^				
Primary Education	44.70	23.81	14.43	< 0.001^*^
Secondary Education	39.67	52.79	56.43	
Tertiary Education	15.63	23.40	28.14	
Employment Status (%)^b^				
Employed	20.40	17.95	15.51	< 0.001^*^
Retired	67.92	48.20	25.76	
Unemployed	11.69	33.85	58.73	
Family economic level (%)^b^				
With great difficulty	12.26	8.41	5.57	< 0.001^*^
With some difficulty	30.68	25.37	22.38	
Fairly easily	33.75	34.72	35.19	
Easily	23.31	31.50	36.86	
Smoke status (%)^b^				
Never smoking	41.63	32.33	26.92	< 0.001^*^
Ever smoker	25.42	28.27	31.64	
Current smoker	32.95	39.40	41.44	
Alcohol intake (%)^b^				
More than recommended level	13.58	21.25	26.50	< 0.001^*^
Heart attack (%)^b^	12.74	8.24	5.31	< 0.001^*^
Hypertension (%)^b^	39.39	32.49	24.54	< 0.001^*^
Hyperlipidemia (%)^b^	24.78	21.75	16.98	< 0.001^*^
Diabetes (%)^b^	14.22	8.76	4.63	< 0.001^*^
Mobility limitation (%)^b^	53.70	32.64	23.52	< 0.001^*^
Physical inactivity (%)^b^	9.54	3.74	2.50	< 0.001^*^
BMI^a^	26.70 ± 4.33	26.56 ± 4.10	26.91 ± 4.71	< 0.001^*^
Cognition^a^	−0.70 ± 3.15	1.11 ± 2.72	2.14 ± 2.54	< 0.001^*^
EURO-D score^c^				
wave 2	1.34 ± 1.07	1.16 ± 1.05	1.10 ± 1.04	< 0.001^*^
wave 4	2.17 ± 1.97	1.73 ± 1.73	1.51 ± 1.61	< 0.001^*^
wave 5	2.24 ± 2.10	1.68 ± 1.78	1.43 ± 1.54	< 0.001^*^
wave 6	2.48 ± 2.24	1.79 ± 1.85	1.56 ± 1.71	< 0.001^*^
wave 7	2.53 ± 2.24	1.83 ± 1.86	1.44 ± 1.58	< 0.001^*^
wave 8	2.52 ± 2.28	1.81 ± 1.85	1.52 ± 1.68	< 0.001^*^

Abbreviations: *BMI* body mass index, *EURO-D* European depression scale

^*^represented *P* value less than 0.05

^a^One-way analysis of variance

^b^Chi-square test

^c^Kruskal-Wallis test

### The joint effect of grip strength and physical inactivity

The results of the multiplicative interaction analysis between grip strength and physical inactivity are presented in Table 2. We also conducted a joint test to assess the association between grip strength, physical inactivity, and depression. A significant interaction effect between grip strength and physical inactivity on depression was identified (joint test: *x*^2^_interaction_ = 11.16, df = 2, *P* = 0.004).

**Table 2:** Interactions between grip strength and physical activity

EURO-D		*β (95%CI)*	*P*
Grip strength	Middle	−0.003(− 0.09–0.08)	0.939
	High	0.03(−0.09–0.15)	0.613
Physical inactivity	Inactivity	0.79(0.61–10.96)	< 0.001^*^
Grip strength × Physical inactivity	Middle × Inactivity	−0.34(− 0.58--0.11)	0.004^*^
	High × Inactivity	−0.47 (− 0.82--0.12)	0.009^*^

Adjusted for age, gender, European region, marital status, education, employment status, family economic level, smoking status, alcohol intake, heart attack, hypertension, hyperlipidemia, diabetes, mobility limitation, body mass index and cognitive function

Abbreviations: *β* coefficient, *CI* confidence interval

^*^represented *P* value less than 0.05

### Impact of physical inactivity according to grip strength groups

**Fig. 2:**
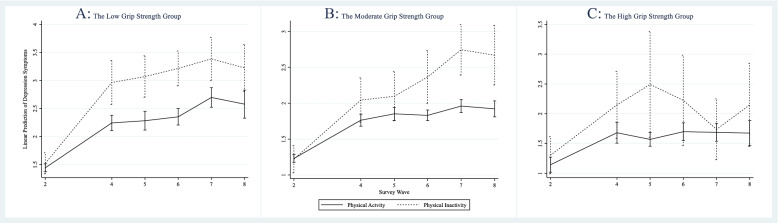
Physical inactivity on longitudinal change of depression symptoms according to grip strength groups

Table 3 summarizes the results of physical inactivity interactions with time according to grip strength groups. Significant interactions between physical inactivity and time with depression were identified in both the low (joint test: *x*^2^_interaction_ = 27.83, df = 4, *P* < 0.001) and moderate (joint test: *x*^2^_interaction_ = 23.67, df = 4, *P* < 0.001) grip strength groups. As shown in Fig. 2A, B, the increasing rate of depression was faster in the physical inactivity group than in the physical activity group. Compared to the physical activity group, participants in the physical inactivity group were positively associated with depression scores at Waves 4 (*β* = 0.64, *P* = 0.002), 5 (*β* = 0.71, *P* = 0.001), 6 (*β* = 0.78, *P* < 0.001), 7 (*β* = 0.61, *P* = 0.009), and 8 (*β* = 0.57, *P* = 0.030) except at baseline (*β* = 0.08, *P* = 0.418) in the low grip strength group. For moderate grip strength, participants in the physical inactivity group had higher depression scores at Waves 6 (*β* = 0.55, *P* = 0.008), 7 (*β* = 0.80, *P* < 0.001), and 8 (*β* = 0.76, *P* = 0.001) but no significant differences were found at baseline (*β* = − 0.01, *P* = 0.913), wave 4 (*β* = 0.29, *P* = 0.124), and wave 5 (*β* = 0.26, *P* = 0.197). No significant interaction between physical inactivity and time with depression was identified in the high (joint test: *x*^2^_interaction_ = 4.39, df = 4, *P* = 0.495) grip strength group (Fig. 2C).

**Table 3:** Interactions between time and physical activity according to grip strength group

Grip strength	EURO-D		*β (95%CI)*	*P*
Low	Time	wave 4	0.80(0.65–0.95)	< 0.001^*^
		wave 5	0.84(0.67–1.01)	< 0.001^*^
		wave 6	0.91(0.75–1.07)	< 0.001^*^
		wave 7	1.25(1.06–1.45)	< 0.001^*^
		wave 8	1.13(0.87–1.40)	< 0.001^*^
	Physical inactivity	Inactivity	0.08(−0.11–0.27)	0.418
	Time × Physical inactivity	wave 4 × Inactivity	0.64(0.24–1.05)	0.002^*^
		wave 5 × Inactivity	0.71(0.28–1.13)	0.001^*^
		wave 6 × Inactivity	0.78(0.40–1.17)	< 0.001^*^
		wave 7 × Inactivity	0.61(0.15–1.06)	0.009
		wave 8 × Inactivity	0.57(0.05–1.08)	0.030^*^
Middle	Time	wave 4	0.53(0.43–0.63)	< 0.001^*^
		wave 5	0.62(0.51–0.73)	< 0.001^*^
		wave 6	0.60(0.51–0.70)	< 0.001^*^
		wave 7	0.73(0.61–0.85)	< 0.001^*^
		wave 8	0.69(0.56–0.83)	< 0.001^*^
	Physical inactivity	Inactivity	−0.01(−0.20–0.18)	0.913
	Time × Physical inactivity	wave 4 × Inactivity	0.29(−0.08–0.66)	0.124
		wave 5 × Inactivity	0.26(−0.13–0.64)	0.197
		wave 6 × Inactivity	0.55(0.14–0.95)	0.008^*^
		wave 7 × Inactivity	0.80(0.39–1.20)	< 0.001^*^
		wave 8 × Inactivity	0.76(0.29–1.23)	0.001^*^
High	Time	wave 4	0.54(0.28–0.80)	< 0.001^*^
		wave 5	0.43(0.24–0.62)	< 0.001^*^
		wave 6	0.55(0.31–0.80)	< 0.001^*^
		wave 7	0.54(0.30–0.78)	< 0.001^*^
		wave 8	0.53(0.22–0.84)	0.001^*^
	Physical inactivity	Inactivity	0.16(−0.17–0.49)	0.345
	Time × Physical inactivity	wave 4 × Inactivity	0.31(−0.33–0.94)	0.345
		wave 5 × Inactivity	0.76(−0.17–1.70)	0.110
		wave 6 × Inactivity	0.37(−0.44–1.17)	0.372
		wave 7 × Inactivity	−0.11(− 0.67–0.45)	0.704
		wave 8 × Inactivity	0.31(−0.43–1.06)	0.409

Adjusted for age, gender, European region, marital status, education, employment status, family economic level, smoking status, alcohol intake, heart attack, hypertension, hyperlipidemia, diabetes, mobility limitation, body mass index and cognitive function

Abbreviations: *β* coefficient, *CI* confidence interval

^*^represented *P* value less than 0.05

### Sensitivity analyses

Both sensitivity analyses recognized low, moderate and high grip strength trajectories according to genders (S**-**Figs. 2 and 3). All trajectories of grip strength met the model evaluation criteria (S**-**Table 6).

The combined effect of grip strength and physical inactivity used wave 2-wave 5 data/ wave 2-wave 7 are shown in S**-**Tables 2 and 4, respectively. Significant interaction effects between grip strength and physical inactivity with depression were found in these two sensitivity analyses (joint test of wave 2–wave 5: *x*^2^_interaction_ = 17.95, df = 2, *P* < 0.001; joint test of wave 2–wave 7: *x*^2^_interaction_ = 16.52, df = 2, *P* < 0.001).

A significant interaction between physical inactivity and time with depression was identified in the low (joint test: *x*^2^_interaction_ = 19.97, df = 2, *P* < 0.001) grip strength group based on wave 2-wave 5 (S**-**Table 3). Significant interactions between physical inactivity and time with depression were identified in both the low (joint test: *x*^2^_interaction_ = 24.95, df = 5, *P* < 0.001) and moderate (joint test: *x*^2^_interaction_ = 23.14, df = 5, *P* < 0.001) grip strength groups based on wave 2-wave 7 (S-Table 5). The influence of physical inactivity on depression over the survey time in different grip strength groups is displayed in S-Figs. 4 and 5, respectively. Sensitivity analyses yielded similar results.

## Discussion

This large longitudinal study has documented two major findings. First, a multiplicative interaction was found between grip strength, physical inactivity, and depressive symptoms. Second, a significant difference in the change in depressive symptoms between physical inactivity and activity was found in the low and moderate grip strength groups, but no significant difference was found in the high grip strength group.

The interaction effect indicated that individuals with physical inactivity and decreased grip strength had a higher risk of depression than those with sufficient physical activity and good grip strength. Previous cohort studies have reported the protective benefits of grip strength in incident depression [9, 29]. The relationship between physical activity and depression have also been well documented. Physical inactivity was related to sustained depression. Physical activity could confer protection against the emergence of depression [30, 31]. The results of the present study support those of the previous studies. Grip strength is commonly used as a measure of muscle strength. Lack of muscular strength may affect myokines released into the circulatory system, which could protect against the risk of depression [32]. In addition, muscular strength is related to sarcopenia [33], functional limitations and disabilities [34]. Individuals with lower grip strength may experience poorer health conditions. A decline in physical function may predict the risk of mental illness [35]. Continuous engagement in physical activity could make the elderly experience more positive leisure activities and sufficient social support, which results in higher psychological well-being and finally reduces depression [36]. However, the associations among grip strength, physical activity, and depression are complex and bidirectional. Depression symptoms are an important mechanism that can impact physical activity [37, 38]. Furthermore, higher physical activity is associated with greater skeletal muscle strength and muscle power [39]. In the present study, our prospective findings indicate that low grip strength and physical inactivity may have a combined effect on depression, both biologically and psychosocially, which is greater than each effect.

Physical inactivity was related to changes in depression in the low and moderate grip strength groups. However, this association was not observed in the high grip strength group. A s British birth cohort study revealed that increased physical activity levels could prevent a decline in grip strength [40]. Randomized controlled trials have shown that physical activity reduces depressive symptoms in older adults [41]. Physical activities can temporarily change central norepinephrine activity, decrease the hypothalamopituitary–adrenocortical axis, and increase the secretion of beta-endorphins, which positively affect mood [42]. In addition, physical activity may increase hippocampal volume and neurogenesis levels, and adjust the imbalance between anti- and pro-inflammatory and oxidant markers to play an antidepressant role [31]. Previous studies have shown that people with depression have lower levels of peripheral brain-derived neurotrophic factor (BDNF), which may contribute to the pathophysiology of depression [43]. Physical activity increases the concentration of several neurotrophic factors, including BDNF, thus possibly exerting a protective effect against depression [44]. Participation in physical activity could give a positive mood to the participant and improve their ability to cope with depression [45]. Compared to the high grip strength group, people in the low- and moderate-strength grip groups were older and more likely to develop chronic diseases. Handgrip strength was an indicator of an individual’s muscle mass, and lower handgrip strength represents poorer health status. Long-term physical inactivity may play an important role in depression in a low-health state. Sufficient physical activity may not prevent depression in participants with health conditions. However, in our present study, the sample size of people with physical inactivity was relatively small, which may have induced an estimated confidence interval that wa too wide resulting in a false negative conclusion.

A major strength of this study was a large number of participants from a prospective study, which provided sufficient power for our statistical analysis. Besides, the GBTM was used to identify grip strength groups by gender based on the study population, which was more suitable than the percentage method. Furthermore, we assessed the association between changes in physical inactivity across the lifespan and depression. However, this study has some limitations. First, self-reported evaluations of physical activity and other health-related statuses might lead to recall bias. Second, physical activity in SHARE did not differentiate between aerobic and strength training, which may have influenced our findings. Second, some participants were excluded due to study criteria. However, most baseline characteristics were well balanced between the included and excluded participants. Finally, despite controlling for many potential covariates, residual confounding may have influenced the observed associations between physical inactivity and grip strength on depression.

## Conclusion

In summary, the results from European middle-aged and older adults indicate that grip strength and physical inactivity have a joint effect on depression. Lower grip strength and physical inactivity can worsen depressive symptoms. Physical inactivity is associated with a change in depression in low and moderate grip strength but not in high grip strength. Therefore, attention should be paid to those with lower grip strength and physical inactivity to prevent depression.

## Supplementary Information

**Additional file 1: S**-**Fig. 1.** Trajectory groups of grip strength according to genders from 2007 to 2019. **S**-**Fig. 2.** Trajectory groups of grip strength according to genders from 2007 to 2011. **S-Fig. 3.** Trajectory groups of grip strength according to genders from 2007 to 2017. **S-Fig. 4.** Physical inactivity on longitudinal change of depression symptoms according to grip strength groups (Wave 2- Wave 5). **S-Fig. 5.** Physical inactivity on longitudinal change of depression symptoms According to grip strength groups (Wave 2- Wave 7).

**Additional file 2: S**-**Table 1.** Characteristics of participants according to analyzed samples. **S**-**Table 2.** Interactions between grip strength and physical activity (Wave 2-Wave 5). **S**-**Table 3.** Interactions between time and physical activity according to grip strength group. **S**-**Table 4**. Interactions between grip strength and physical activity (Wave 2-Wave 7). **S**-**Table 5.** Interactions between time and physical activity according to grip strength group (Wave 2-Wave 7). **S**-**Table 6**. Model evaluation indexes of grip strength trajectories in both genders according to different survey wave.

## Data Availability

The data that support the findings of this study are available in the Survey of Health, Ageing and Retirement in Europe at doi:10.1093/ije/dyt088. These data were derived from the following resources available in the public domain: http://www.share-project.org/data-access.html.

## Abbreviation

SHARE: Survey of Health, Ageing and Retirement in Europe
GBTM: Group-based trajectory modeling
EURO-D: European depression scale
SD: Standard deviation
ANOVA: One-way analysis of variance
GEE: Generalized estimated equation
BDNF: Brain-derived neurotrophic factor
