# Supplementary material for: Lower grip strength and insufficient physical activity can increase depressive symptoms among middle-aged and older European adults: a longitudinal study
Source: BMC Geriatr. 2022 Aug 22;22:696. doi: 10.1186/s12877-022-03392-x (PMC9396791; doi:10.1186/s12877-022-03392-x)
Supplement: Supplementary file 1 — Additional file 1: S-Fig. 1. Trajectory groups of grip strength according to genders from 2007 to 2019. S-Fig. 2. Trajectory groups of grip strength according to genders from 2007 to 2011. S-Fig. 3. Trajectory groups of grip strength according to genders from 2007 to 2017. S-Fig. 4. Physical inactivity on longitudinal change of depression symptoms according to grip strength groups (Wave 2- Wave 5). S-Fig. 5. Physical inactivity on longitudinal change of depression symptoms According to grip strength groups (Wave 2- Wave 7). [file 12877_2022_3392_MOESM1_ESM.docx]

**S-Figure 1.** Trajectory groups of grip strength according to genders from 2007 to 2019

**S-Figure 2.** Trajectory groups of grip strength according to genders from 2007 to 2011

**S-Figure 3.** Trajectory groups of grip strength according to genders from 2007 to 2017

**S-Figure 4**. Physical inactivity on longitudinal change of depression symptoms According to grip strength groups (Wave 2- Wave 5)

**S-Figure 5**. Physical inactivity on longitudinal change of depression symptoms According to grip strength groups (Wave 2- Wave 7)

**The caption of Supplementary Figure 1 to 3:**

Trajectory groups of grip strength by gender were estimated using group-based trajectory modeling (GBTM). GBTM was developed to identify the optimal number of distinct groups that classify individuals according to their longitudinal pattern. GBTM identified groupings of individuals incorporating all the data points instead of a single point of time. Considering the difference in grip strength between males and females, GBTMs were conducted as gender-specific.
