# Supplementary material for: Lower grip strength and insufficient physical activity can increase depressive symptoms among middle-aged and older European adults: a longitudinal study
Source: BMC Geriatr. 2022 Aug 22;22:696. doi: 10.1186/s12877-022-03392-x (PMC9396791; doi:10.1186/s12877-022-03392-x)
Supplement: Supplementary file 2 — Additional file 2: S-Table 1. Characteristics of participants according to analyzed samples. S-Table 2. Interactions between grip strength and physical activity (Wave 2-Wave 5). S-Table 3. Interactions between time and physical activity according to grip strength group. S-Table 4. Interactions between grip strength and physical activity (Wave 2-Wave 7). S-Table 5. Interactions between time and physical activity according to grip strength group (Wave 2-Wave 7). S-Table 6. Model evaluation indexes of grip strength trajectories in both genders according to different survey wave. [file 12877_2022_3392_MOESM2_ESM.docx]

**S-Table 1**. Characteristics of participants according to analyzed samples

|  | Original Wave 2 (n=25083) | Final Analyzed Wave 2 (n=14098) | *P* |
| --- | --- | --- | --- |
| Age ^a^ | 64.65±9.33 | 63.62±8.40 | <0.001^*^ |
| Gender (%) ^b^ |  |  | 0.038^*^ |
| Female | 49.70 | 50.79 |  |
| Region (%) ^b^ |  |  | <0.001^*^ |
| Central Europe | 34.23 | 31.86 |  |
| Northern Europe | 16.75 | 17.97 |  |
| Southern Europe | 22.34 | 25.67 |  |
| Western Europe | 26.68 | 24.51 |  |
| Married Status (%) ^b^ |  |  | 0.004^*^ |
| Married, living with a spouse | 74.42 | 75.73 |  |
| Education (%) ^b^ |  |  | 0.006^*^ |
| Primary Education | 26.62 | 26.10 |  |
| Secondary Education | 51.67 | 50.79 |  |
| Tertiary Education | 21.72 | 23.11 |  |
| Employment Status (%) ^b^ |  |  | <0.001^*^ |
| Employed | 17.65 | 17.88 |  |
| Retired | 50.07 | 46.96 |  |
| Unemployed | 32.28 | 35.15 |  |
| Family economic level (%) ^b^ |  |  | 0.128^*^ |
| With great difficulty | 8.60 | 8.55 |  |
| With some difficulty | 26.18 | 25.80 |  |
| Fairly easily | 35.33 | 34.62 |  |
| Easily | 29.89 | 31.03 |  |
| Smoke status (%) ^b^ |  |  | <0.001^*^ |
| Never smoking | 29.42 | 33.04 |  |
| Ever smoker | 32.89 | 28.48 |  |
| Current smoker | 37.69 | 38.48 |  |
| Alcohol intake (%) ^b^ |  |  | 0.768 |
| More than recommended level | 20.74 | 20.86 |  |
| Heart attack (%) ^b^ | 10.33 | 8.50 | <0.001^*^ |
| Hypertension (%) ^b^ | 33.01 | 32.03 | 0.049^*^ |
| Hyperlipidemia (%) ^b^ | 20.51 | 21.23 | 0.091 |
| Diabetes (%) ^b^ | 9.46 | 8.94 | 0.084 |
| Mobility limitation (%) ^b^ | 38.63 | 35.03 | <0.001^*^ |
| Physical inactivity (%) ^b^ | 6.30 | 4.72 | <0.001^*^ |
| BMI ^a^ | 26.62±4.33 | 26.68±4.32 | <0.001^*^ |
| Cognition ^a^ | 0.65±3.12 | 0.97±2.95 | <0.001^*^ |
| EURO-D score ^c^ | 1.20±1.07 | 1.19±1.06 | 0.084 |

Abbreviations: BMI, body mass index; EURO-D, European depression scale

^*^ represented *P* value less than 0.05

^a^ Z-test

^b^ Chi-square test

^c^ Wilcoxon rank-sum test

**S-Table 2**. Interactions between grip strength and physical activity (Wave 2-Wave 5)

| EURO-D | | *β (95%CI)* | *P* |
| --- | --- | --- | --- |
| Grip strength | Middle | -0.03(-0.13-0.07） | 0.549 |
|  | High | 0.003(-0.13-0.14) | 0.933 |
| Physical inactivity | Inactivity | 0.88(0.59-1.17) | <0.001^*^ |
| Grip strength × | Middle × Inactivity | -0.74(-1.09--0.40) | <0.001^*^ |
| Physical inactivity | High × Inactivity | -0.47(-0.96--0.02) | 0.062 |

Abbreviations: *β*, coefficient, *CI*, confidence interval

^*^ represented *P* value less than 0.05

Adjusted for age, gender, European region, marital status, education, employment status, family economic level, smoking status, alcohol intake, heart attack, hypertension, hyperlipidemia, diabetes, mobility limitation, body mass index and cognitive function

**S-Table 3**. Interactions between time and physical activity according to grip strength group

(Wave 2-Wave 5)

| Grip strength | EURO-D | | *β (95%CI)* | *P* |
| --- | --- | --- | --- | --- |
| Low | Time | wave 4 | 0.62(0.43-0.82) | <0.001^*^ |
|  |  | wave 5 | 0.84(0.60-1.08) | <0.001^*^ |
|  | Physical inactivity | Inactivity | 0.06(-0.20-0.32) | 0.661 |
|  | Time × Physical inactivity | wave 4×Inactivity | 0.85(0.36-1.35) | 0.001^*^ |
|  |  | wave 5×Inactivity | 0.93(0.42-1.45) | <0.001^*^ |
| Middle | Time | wave 4 | 0.46(0.36-0.57) | <0.001^*^ |
|  |  | wave 5 | 0.59(0.47-0.70) | <0.001^*^ |
|  | Physical inactivity | Inactivity | 0.02(-0.19-0.24) | 0.825 |
|  | Time × Physical inactivity | wave 4×Inactivity | 0.07(-0.31-0.44) | 0.730 |
|  |  | wave 5×Inactivity | 0.28(-0.11-0.67) | 0.158 |
| High | Time | wave 4 | 0.53(0.29-0.77) | <0.001^*^ |
|  |  | wave 5 | 0.48(0.31-0.64) | <0.001^*^ |
|  | Physical inactivity | Inactivity | 0.07(-0.23-0.37) | 0.660 |
|  | Time × Physical inactivity | wave 4×Inactivity | 0.25(-0.34-0.84) | 0.406 |
|  |  | wave 5×Inactivity | 0.90(-0.02-1.81) | 0.055 |

Abbreviations: *β*, coefficient, *CI*, confidence interval

^*^ represented *P* value less than 0.05

Adjusted for age, gender, European region, marital status, education, employment status, family economic level, smoking status, alcohol intake, heart attack, hypertension, hyperlipidemia, diabetes, mobility limitation, body mass index and cognitive function

**S-Table 4**. Interactions between grip strength and physical activity (Wave 2-Wave 7)

| EURO-D | | *β (95%CI)* | *P* |
| --- | --- | --- | --- |
| Grip strength | Middle | -0.005(-0.09-0.08） | 0.909 |
|  | High | 0.05(-0.07-0.16) | 0.438 |
| Physical inactivity | Inactivity | 0.84(0.65-1.03) | <0.001^*^ |
| Grip strength × | Middle × Inactivity | -0.45(-0.69--0.20) | <0.001^*^ |
| Physical inactivity | High × Inactivity | -0.59(-0.96--0.23) | 0.001^*^ |

Abbreviations: *β*, coefficient, *CI*, confidence interval

^*^ represented *P* value less than 0.05

Adjusted for age, gender, European region, marital status, education, employment status, family economic level, smoking status, alcohol intake, heart attack, hypertension, hyperlipidemia, diabetes, mobility limitation, body mass index and cognitive function

**S-Table 5**. Interactions between time and physical activity according to grip strength group

(Wave 2-Wave 7)

| Grip strength | EURO-D | | *β (95%CI)* | *P* |
| --- | --- | --- | --- | --- |
| Low | Time | wave 4 | 0.81(0.65-0.98) | <0.001^*^ |
|  |  | wave 5 | 0.88(0.68-1.08) | <0.001^*^ |
|  |  | wave 6 | 0.94(0.77-1.11) | <0.001^*^ |
|  |  | wave 7 | 1.32(1.11-1.54) | <0.001^*^ |
|  | Physical inactivity | Inactivity | 0.12(-0.08-0.32) | 0.252 |
|  | Time × Physical inactivity | wave 4×Inactivity | 0.67(0.25-1.09) | 0.002^*^ |
|  |  | wave 5×Inactivity | 0.73(0.29-1.18) | 0.001^*^ |
|  |  | wave 6×Inactivity | 0.81(0.39-1.22) | <0.001^*^ |
|  |  | wave 7×Inactivity | 0.50(0.02-0.97) | 0.041^*^ |
| Middle | Time | wave 4 | 0.53(0.43-0.62) | <0.001^*^ |
|  |  | wave 5 | 0.61(0.50-0.71) | <0.001^*^ |
|  |  | wave 6 | 0.58(0.49-0.68) | <0.001^*^ |
|  |  | wave 7 | 0.70(0.59-0.80) | <0.001^*^ |
|  | Physical inactivity | Inactivity | -0.02(-0.21-0.16) | 0.808 |
|  | Time × Physical inactivity | wave 4×Inactivity | 0.30(-0.05-0.66) | 0.092 |
|  |  | wave 5×Inactivity | 0.27(-0.11-0.64) | 0.164 |
|  |  | wave 6×Inactivity | 0.57(0.19-0.95) | 0.004^*^ |
|  |  | wave 7×Inactivity | 0.87(0.48-1.27) | <0.001^*^ |
| High | Time | wave 4 | 0.55(0.30-0.80) | <0.001^*^ |
|  |  | wave 5 | 0.46(0.28-0.64) | <0.001^*^ |
|  |  | wave 6 | 0.58(0.33-0.82) | <0.001^*^ |
|  |  | wave 7 | 0.60(0.36-0.84) | <0.001^*^ |
|  | Physical inactivity | Inactivity | 0.15(-0.18-0.47) | 0.370 |
|  | Time × Physical inactivity | wave 4×Inactivity | 0.22(-0.37-0.81) | 0.457 |
|  |  | wave 5×Inactivity | 0.69(-0.24-1.63) | 0.148 |
|  |  | wave 6×Inactivity | 0.22(-0.54-0.98) | 0.567 |
|  |  | wave 7×Inactivity | -0.11(-0.71-0.48) | 0.716 |

Abbreviations: *β*, coefficient, *CI*, confidence interval

^*^ represented *P* value less than 0.05

Adjusted for age, gender, European region, marital status, education, employment status, family economic level, smoking status, alcohol intake, heart attack, hypertension, hyperlipidemia, diabetes, mobility limitation, body mass index and cognitive function

**S-Table 6**. Model evaluation indexes of grip strength trajectories in both genders according to different survey wave

| Survey Wave | Gender | Grip strength trajectory | BIC | APPA | OCC |
| --- | --- | --- | --- | --- | --- |
| Wave 2-Wave 5 | Male | Low | -23469.90 | 0.900 | 86.558 |
|  |  | Moderate |  | 0.887 | 21.473 |
|  |  | High |  | 0.902 | 50.306 |
|  | Female | Low | -20318.75 | 0.886 | 60.151 |
|  |  | Moderate |  | 0.891 | 23.286 |
|  |  | High |  | 0.903 | 76.822 |
| Wave 2-Wave 7 | Male | Low | -46473.65 | 0.907 | 83.731 |
|  |  | Moderate |  | 0.904 | 25.127 |
|  |  | High |  | 0.912 | 62.925 |
|  | Female | Low | -39388.71 | 0.897 | 64.519 |
|  |  | Moderate |  | 0.894 | 25.309 |
|  |  | High |  | 0.912 | 82.211 |
| Wave 2-Wave 8 | Male | Low | -51707.01 | 0.911 | 83.352 |
|  |  | Moderate |  | 0.904 | 25.684 |
|  |  | High |  | 0.915 | 65.309 |
|  | Female | Low | -43831.31 | 0.904 | 62.992 |
|  |  | Moderate |  | 0.899 | 27.562 |
|  |  | High |  | 0.914 | 89.547 |

Abbreviations: BIC, Bayesian information criterion; APPA, average posterior probability of assignments; OCC, odds of correct classification
